# Characterization of Volatile and Flavonoid Composition of Different Cuts of Dried Onion (*Allium cepa* L.) by HS-SPME-GC-MS, HS-SPME-GC×GC-TOF and HPLC-DAD

**DOI:** 10.3390/molecules25020408

**Published:** 2020-01-18

**Authors:** Lorenzo Cecchi, Francesca Ieri, Pamela Vignolini, Nadia Mulinacci, Annalisa Romani

**Affiliations:** 1 Department of NEUROFARBA, University of Florence, Via Ugo Schiff, 6, 50019 Sesto Fiorentino FI, Florence, Italy; lo.cecchi@unifi.it (L.C.); nadia.mulinacci@unifi.it (N.M.); 2 QuMAP Laboratory, PIN Polo Universitario Città di Prato, Piazza Giovanni Ciardi, 25, 59100 Prato PO, Italy; annalisa.romani@unifi.it; 3 Department of Statistic, Informatics and Applications “G. Parenti” (DiSIA)—University of Florence, Phytolab Laboratory, via Ugo Schiff 6, 50019 Sesto Fiorentino FI, Italy; pamela.vignolini@unifi.it

**Keywords:** volatile compounds, onion rings, onion flakes, phenolic compounds, flavonoids, sulfur compounds, food ingredient

## Abstract

Onion is widely used worldwide in various forms for both food and medicinal applications, thanks to its high content of phytonutrients, such as flavonoids and volatile sulfur compounds. Fresh onion is very perishable and drying is widely applied for extending shelf-life, thus obtaining a very easy-to-use functional food ingredient. The flavonoid and volatile fractions of different onion cuts (flakes, rings) prepared through different drying cycles in a static oven, were characterized by high-performance liquid chromatography with a diode-array detector HPLC-DAD, Head Space-Solid Phase Micro Extraction followed by Gas Chromatography coupled with Mass Spectrometry (HS-SPME-GC-MS) and Head-Space Solid Phase Micro Extraction followed by comprehensive two-dimensional Gas-Chromatography (HS-SPME-GC×GC-TOF). Onion flakes showed a significantly higher flavonoid content (3.56 mg g^−1^) than onion rings (2.04 mg g^−1^). Onion flakes showed greater amount of volatile organic compounds (VOCs) (127.26 mg g^−1^) than onion rings (42.79 mg g^−1^), with different relative amounts of di- and trisulfides—disulfides largely predominate the volatile fraction (amounts over 60% on the total volatile content), followed by trisulfides and dipropyl disulfide and dipropyl trisulfide were the most abundant VOCs. HS-SPME-GC×GC-TOF allowed for the detection of the presence of allylthiol, diethanol sulfide, 4,6-diethyl1,2,3,5-tetrathiolane, not detected by HS-SPME-GC-MS, and provided a fast and direct visualization and comparison of different samples. These results highlight different nutraceutical properties of dried onion samples processed otherwise, only differing in shape and size, thus pointing out potentially different uses as functional ingredients.

## 1. Introduction

*Allium* is the larger genus of the Alliaceae family, with approx. 450 species [1]. Among these species, onion (*Allium cepa* L.) is widely used in various forms all over the world as part of the international cuisine, in food processing but also in traditional medicine [2]. It has been cultivated for more than 4000 years, with USA, China, Turkey, Russia and Egypt as the main producers [3]. Some of the reported properties of onion are antidiabetic, antibiotic, antioxidant, antimicrobial, antifungal, antiasthmatic, anticancer, anti-inflammatory, hypolipidemic and anticholesterolemic [2,4,5,6,7,8] but also the usefulness of consuming dried onions in preventing viral infections, coronary hearth diseases, cataract and disturbances of the gastrointestinal tract have been described [1,9]. Fresh onion is very perishable and the postharvest action of enzymes as pectin methylesterase and polygalatturonase lead to onion softening [10], thus, in order to extend onion shelf life, drying fresh onion for obtaining chip or powder is one of the most used processes [11,12]; dried onion has gained increasingly attention as an alternative to the fresh onion as an ingredient for sauces, snacks and frozen foods, for both industrial and domestic uses, also thanks to several characteristics meeting the consumers’ expectations [13,14]. In the powdered form, dried onion could be a very easy-to-use functional food ingredient [12]. In 2017, according to Food and Agriculture Organization (FAO), the global production of dried onion accounted for about 97 million tons, with China and India as the main producers, while the Italian production accounted for approx. 0.4 million tons (≈0.4% of the global production).

The characteristic flavor and biological properties at the basis of these uses of onion have been mainly attributed to the sulfur compounds present in the volatile fraction of onion. The volatile compounds emitted by onion also demonstrated anti-browning activity, with trisulfides as better inhibitors than disulfides [4,15]. It has been reported that the antimicrobial activity of sulfides is affected by the number of *C*-atoms of the alk(en)yl groups and *S*-atoms and by the concentration of the different sulfur compounds in the volatile profile of onion, pointing out the importance of characterizing the volatile profile of onion in the different used forms, in that the composition of the sulfur compounds varies among the different flavored forms of onions [4,12,14]. Another class of volatile compounds detected in fresh or treated onion is that of aldehydes [12,14].

**Figure 1:**
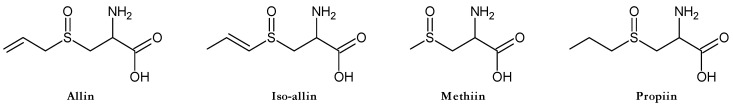
Typical *S*-alk(en)yl-L-cysteine-*S*-oxide found in different *Allium* species.

The type of sulfur compounds found in onions and other *Allium* species is strongly affected by the nature of the *S*-alk(en)yl-L-cysteine-*S*-oxide precursors (Figure 1). For example, in garlic, propiin is almost absent and alliin is the major compound, while in onion, alliin is present at a very low concentration [16] and iso-alliin is the major metabolite. Iso-allin and its derivatives containing the 1-propenyl moiety are usually present as a mixture of the *E* and *Z* isomers. In onion, some derivatives of iso-alliin are also transformed in *Z*-propanethial *S*-oxide (the lachrymatory factor) and its isomer *E*-propanethial *S*-oxide, *cis* and *trans*-zwiebelane and cepaenes [1], which largely predominate in fresh onion.

Analysis by SPME-GC-MS of fresh, frozen, freeze-dried and sterilized onions pointed out that, in the transformed samples, most of the sulfur compounds present in fresh onion (thiosulfinates and zwiebelanes) degraded to form other sulfur volatiles—mainly disulfides and trisulfides—with the consequent changes in sensorial and nutraceuticals properties [17]. For example, frozen onions were reported to be rich in 2 methyl-2-pentenal (a degradation product of the LF) and three disulfides, while in dried onions a strong diminution of disulfides, a certain increase of some trisulfides and the presence of tetrasulfides were reported [12,17].

Besides volatile sulfur compounds, other polar compounds, as sapogenins, saponins and flavonoids have been detected in the *Allium* species [1]. Several flavonoids, whose nutraceutical properties are well-known, have been identified in fresh onion, with quercetin-4′-*O*-monoglucoside and quercetin-3,4′-*O*-diglucoside as the most abundant ones [18].

In this study, we aimed at characterizing samples prepared by different cuts (onion flakes and onion rings) and different drying cycles in a static oven of dried white onion from the Emilia Romagna region (Italy). We hypothesized that the different cuts of onion would show different chemical composition, thus providing different nutraceutical properties. The flavonoid fraction was characterized by HPLC-DAD. The volatile fraction was analyzed using optimized Head Space-Solid Phase Micro Extraction followed by Gas Chromatography coupled with Mass Spectrometry (HS-SPME-GC-MS); in addition, Head-Space Solid Phase Micro Extraction followed by comprehensive two-dimensional Gas-Chromatography (HS-SPME-GC×GC-TOF) was adopted for providing a volatile fingerprint of the two samples for a fast and direct visualization and comparison.

## 2. Results and Discussion

Two different kinds of dried onion samples were obtained by drying different cuts (onion rings and onion flakes) of Emilia Romagna’s white onion (*Allium cepa* L.) in a static oven at 40 °C. The chemical composition of the two samples was analyzed in order to point out any variances between them and any consequent different possible nutraceutical application of these products. The volatile and flavonoid fractions of the samples were analyzed using chromatographic techniques (HPLC-DAD, HS-SPME-GC-MS and HS-SPME-GC×GC-TOF), while the total phenolic content was evaluated using the Folin-Ciocalteu method. HS-SPME was used as a well-recognized convenient sampling tool for VOCs, coupled with GC-MS for their separation and detection, techniques which are increasingly applied for analysis of foods. HS-SPME-GC×GC-TOF was thereafter applied for deep-in elucidation of the volatile profile of samples. To the author knowledge, this paper is the first report on the application of HS-SPME-GC×GC-TOF to the analysis of the volatile fraction of onion samples.

### 2.1. Volatile Characterization

#### 2.1.1. HS-SPME-GC-MS Analysis

Table 1 shows the composition of the volatile profile of the dried onion samples when they were analyzed by HS-SPME-GC-MS either in the presence or in the absence of ascorbic acid. Overall, a total of 53 volatile organic compounds (VOCs) were tentatively identified, according to their RI and matching factor greater than 80%—30 of them are sulfur-containing compounds (3 monosulfides, 16 disulfides, 5 trisulfides, 1 mercaptane, 2 thiophenes, 2 trithiolanes and the carbon disulfide), while the remaining 23 VOCs were 15 aldehydes, 1 ketone, 3 carboxylic acids, 1 alcohol, 2 esters and the 2-pentylfuran. The reported data in Table 1 are based on the use of the internal standard, namely 4-methyl-2-pentanol and they have to be intended as a relative quantitation. The total content of VOCs detected in dried onion flakes is much higher than in dried onion rings, with values of 127.26 mg g^−1^ in onion flakes, which is the triple of the 42.79 mg g^−1^ in onion rings.

Sulfides—Disulfides are the most abundant class of VOCs in the volatile fraction of the samples followed by trisulfides. These two classes, together, account for the 92.6% of the total VOCs content in onion flakes and 89.2% in onion rings. In particular, the class of disulfides accounts for the 60.3% in onion flakes and for 76.9% in onion rings, with dipropyl disulfide as the definitely most abundant molecule of this class, followed by propyl *trans*-1-propenyl disulfide, methyl propyl disulfide, methyl cis-1-propenyl disulfide and methyl *trans*-1-propenyl disulfide; the dipropyl disulfide, identified in our samples as the most abundant VOC, was previously reported as linked to green notes of onion. At the same time, the class of trisulfides accounts for 32.3% in onion flakes and for 12.4% in onion rings, with dipropyl trisulfide as the most abundant molecule of this class, followed by dimethyl trisulfide, methyl propyl trisulfide and propyl *trans*-1-propenyl trisulfide. The presence of disulfides and trisulfides in so high percentages in the volatile profile of these dried onion samples is in agreement with previous literature [1,17], in which the authors stated that frozen onion maintains a profile of VOCs similar to fresh onion, while other transformed onion samples, as dried onion, show the presence of high amounts of di- and trisulfides. Monosulfides were detected in very low amounts (0.32 mg g^−1^ in onion flakes and 0.09 mg g^−1^ in onion rings), with allyl propyl sulfide as most abundant one in both the samples. To the authors’ knowledge, some of the sulfides detected in low amount in this research have never been reported before in the volatile fraction of dried onion samples—allyl propyl sulfide, 1-propenyl propyl sulfide, allyl isopropyl disulfide, allyl cis-1-propenyl disulfide, allyl *trans*-1-propenyl disulfide, 1-(1-(methylthio)propyl)-2-propyl disulfide, methyl 1-(propylthio)propyl disulfide, 1-(cis-1-propenylthio)propyl propyl disulfide, 1-(1-*trans*-propenylthio)propyl propyl disulfide.

Other S-compounds—a total of 6 S-containing VOCs other than sulfides were identified, for a total content of 6.08 mg g^−1^ in onion flakes and 2.45 mg g^−1^ in onion rings. Dimethylthiophenes and 3,5-diethyl-1,2,4-trithiolanes were present in significant concentration. In particular, the presence of dialkylthiophenes in the volatile profile of onion has been previously reported as being due to the thermolysis of alkyl 1-propenyl disulfides and di(1-alkenyl)disulfides and in a previous study in which fresh onion was dried at different temperatures, their amount increased the most when the drying temperature was the highest [12]. The total content of dialkylthiophenes in our samples is in low percentages, ranging 3%–4%; this datum, compared with those reported by Choi et al., 2017 [12] confirms that the low temperature of the applied drying process allowed a partial preservation of the volatile profile of onion samples and in particular the disulfides.

In order to detect any oxidation process affecting the composition of S-containing molecules in the applied analytical condition and to verify if the presence of an anti-oxidant molecule could inhibit these processes, further analysis was carried out adding ascorbic acid, a molecule well-known for its anti-oxidant properties, to the sample. When samples were analyzed in the presence of ascorbic acid, the total VOCs content increased from 42.79 mg g^−1^ to 65.42 mg g^−1^ in onion rings (+22.63 mg g^−1^, +52.8%) and from 127.26 mg g^−1^ to 136.21 mg g^−1^ in onion flakes (+8.95 mg g^−1^, +7.0%). This different behavior of the two samples was mainly due to the sulfides—in fact, for the onion flakes none of the VOCs in the classes of mono-, di- and trisulfides showed significant differences between the sample analyzed in the presence or in the absence of ascorbic acid, while for onion rings, the content of sulfides was higher in the presence of ascorbic acid. However, the type of sulfur-containing compounds is the same in the presence or in the absence of ascorbic acid. Some other experiments, out of the aims of this work, should be performed to clarify the reasons behind the different behavior of the presence of ascorbic acid in the two samples. Our data suggest that the addition of this antioxidant molecule during analysis of a dried onion sample does not provide different qualitative (and presumably also quantitative) profiles of sulfur-containing compounds, which consequently seem not to be affected by the analytical conditions. We can thus hypothesize that, in the dried onion samples, nonoxidized volatile S-compounds as thiols are absent or present in negligible amounts, in that already transformed in the oxidized forms of di- or trisulfides.

Regarding sulfides, Table 2 shows the sum of the relative concentration of the molecules containing the 1-propenyl or the allyl moiety. According to the higher content of iso-alliin than alliin in onion, the concentration of VOCs with the 1-propenyl moiety was higher than those with the allyl moiety; however, the presence of allyl derivatives also suggests the presence of low amounts of alliin, whose transformation after onion cutting leads to allyl derivatives. The detection of certain amounts of allyl derivatives in onion processed product is in agreement with previous literature [20]

Aldehydes—a total of 16 aldehydes were identified in the volatile profile of the dried onion samples, 4 of which never reported in this type of samples so far (2-methyl propanal, pentanal, octanal and furfural). The total content of aldehydes was slightly higher in dried onion flakes (1.80 µg g^−1^) than in dried onion rings (1.31 µg g^−1^) and the content was higher in both the samples when they were analyzed in the presence of ascorbic acid. This higher content in the presence of ascorbic acid was almost completely due to octanal, furfural and decanal, absent or present in negligible amounts when the samples were analyzed in the absence of ascorbic acid and present in significant amount when the samples were analyzed in the presence of ascorbic acid—these three aldehydes were not found in a previous study analyzing dried onion samples [12]. If furfural is a well-known degradation products of ascorbic acid [21], our data pointed out that the presence of ascorbic acid affects the content of octanal and decanal in the volatile profile of dried onion samples. We can hypothesize that the antioxidant activity of ascorbic acid prevents further oxidation of these molecules in the analytical conditions but further investigation should also be performed in future studies to exclude that also these 2 aldehydes are degradation products of ascorbic acid. Regarding the content of the different aldehydes in the samples analyzed in the absence of ascorbic acid, nonanal was only present in trace, while the greatest content was for 3-methyl butanal in dried onion flakes (0.36 µg g^−1^). The content of the most of the identified aldehydes did not significantly differ between dried onion flakes and dried onion rings. The content of the following 5 aldehydes was significantly different and higher in onion flakes—propanal, hexanal, benzaldehyde, Z-2-heptenal and 2-methyl-2-pentenal, with this latter molecule that was reported to be the main degradation product of the lachrymatory factor [14]. The presence of high amounts of hexanal and 2-methyl-2-pentenal was recently associated to some off-flavor attributes of onion powder, as humidity [12]; our results show low amount of these two aldehydes (approx. 0.2% of the total VOCs content), this result pointing out that in the analyzed sample this off-flavor can be considered absent.

Other VOCs—6 volatile compounds other than aldehydes or S-containing compounds were detected in our samples in low amounts—6-methyl-5-hepten-2-one, nonanoic acid, 2-pentylfuran, 1-octen-3-ol, isopropyl dodecanoate and 2,2,4-trimethyl-1,3-pentanediol diisobutyrate. These VOCs showed slight differences between onion flakes and onion rings and their amount did not change when the samples were analyzed in the presence of ascorbic acid. The only exception was the nonanoic acid, which content strongly increased in the presence of ascorbic acid, which also led to the detection of hexanoic and 2-ethyl hexanoic acids.

#### 2.1.2. HS-SPME-GC×GC-TOF Analysis

HS-SPME-GC×GC-TOF analysis was applied to better elucidate the volatile profile, thus providing a tool for the direct comparison and visualization of volatile components and pointing out the presence of molecules not identified only with GC-MS. HS-SPME-GC×GC-TOF analyses of the volatile fraction of onion products were submitted to advanced fingerprinting analysis of 2D chromatographic data.

Sulfur-containing compounds were the most abundant in samples volatile fraction. Sulfur compounds are considered to be derived from the degradation of sulfur-containing amino-acids and are associated with alliaceous, sulfuric, sweaty, onion and cabbage aromas, contributing to the characteristic flavor of raw and processed edible *Alliums*. Table 3 reports the list of headspace volatile compounds of the onion products, 1st D and 2nd D retention times and the average peak volume from three independent determination. A split ratio of 1:5 during SPME injection was employed for a better separation in the 2D chromatographic space of the major co-eluting molecules not evidenced in the common 1D analysis. The most intense peaks corresponded to dipropyl disulfide and dipropyl trisulfide. Diethanol disulfide and allylthiol were identified only by GC×GC, probably owing to the co-elution with peaks deriving from SPME fiber bleeding and/or other molecules in mono-dimensional chromatography. Also peaks of *cis*-3,5-diethyl-1,2,4-trithiolane and *trans*-3,5-diethyl-1,2,4-trithiolane (which probably is a peak constituted by the two co-eluting enantiomers) were simultaneous separated by GC×GC, in particular the *trans* and *cis* isomers were separated in the first dimension (29.900, 1.620 min and 30.150, 1.640 min, respectively). Another trisulfide, the propenyl propyl trisulfide, was separated in the second dimension, eluting at 30.150, 1.380 min. Tentative identification was made on the basis of non-isothermal Kovats retention indices from temperature-programming from Chemistry WebBook (Table 1) and on the presence of the ion 151 which was present only in cyclic trisulfides (*cis* and *trans* 3,5-diethyl-1,2,4-trithiolane) and not in the linear ones (propenyl propyl trisulfide). Trithiolane compounds have been identified as important aroma-active compounds in cooked onions (*Allium cepa* L.) showing cooked onion-like/fruity and blackcurrant-like/fruity odor impressions for the *trans*-enantiomers compared to the *cis*-isomer eliciting a meat broth-like, cooked onion-like aroma at a five- to ten-fold higher threshold [22].

**Figure 2:**
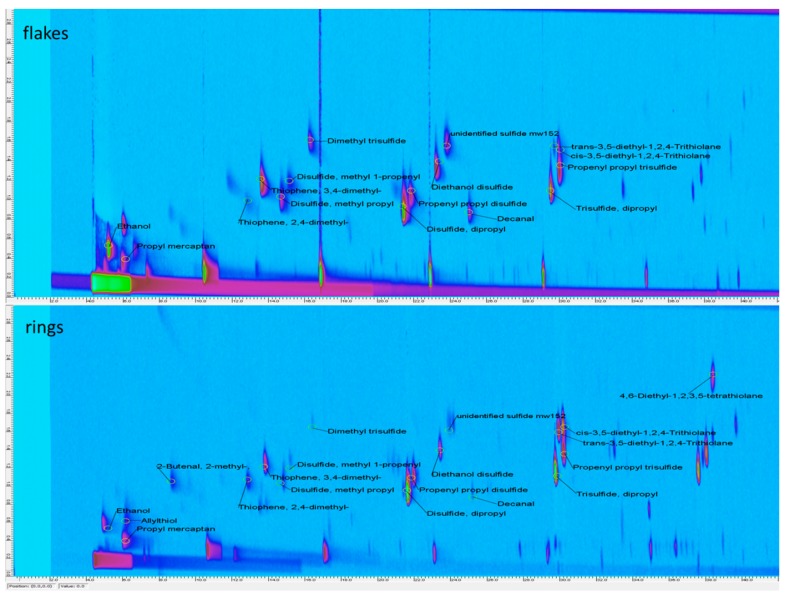
Comprehensive two-dimensional chromatography–mass spectrometry (GC×GC-TOF) color diagram and comprehensive template matching fingerprinting with the main identified volatile compounds of onion flakes and rings.

In Figure 2, “contour plots” from HS-SPME-GC×GC-TOF analyses of the onion products are reported—each 2D-peak corresponds to a single volatile compound. In this case, SPME and comprehensive comparative analysis of 2D chromatographic data showed visual differences between samples. The main differences that emerged between the two samples could be summarized as follows—flakes showed higher intensity of sulfides peaks as reported by GC-MS; allylthiol, 2-methyl-butenal and 4,6-diethyl-1,2,3,5-tetrathiolane were detected only in rings sample.

### 2.2. Phenolic Characterization

Data reported in literature for onion phenolic composition vary due to normal biological variations related to cultivar, growing season, environmental and agronomic conditions and, in some instances, the region of the bulb [1,23].

HPLC analysis of our hydroalcoholic onion extracts produced two principal peaks identified as quercetin-3,4′-diglucoside and quercetin-4-glucoside by comparison of spectroscopic and literature data; they are the main compounds in our sample, data in agreement with the literature [24,25,26]. Their content (Table 4) was higher than that reported in recent researches [1,23] and, on percentage, ranges between 45.8%–49.3% and 42.4%–42.8%, respectively. The sum of the content of these two molecules accounts for 92% of the total flavonoid content in onion flakes and 88% of the total flavonoid content in onion rings, values even higher than the 80% [27] or 85% [18] and in agreement with the 93% [28] previously reported. On the contrary, quercetin was present in percentage ranging 1%–2% on the total flavonoid content, again in agreement with the previous literature [18,29].

Considering that in fresh onion the content of flavonoids ranges from 0.185 to 0.634 mg g^−1^ and that approx. 90% of fresh onion is constituted by water, our data suggest that the applied drying process leads to only a slight loss of flavonoid, which thus resulted in a concentration approx. 10 times that of the fresh onion.

Further analysis was performed to evaluate the total polyphenol content—the values ranged from 1.49 (onion rings) to 1.62 mg_gallic acid_ g^−1^ (onion flakes), data in agreement to Manohar et al. 2017 [30] but lower than those reported in another recent study [31]. The difference in this latter study could be due to the lower drying temperature applied in our study—indeed, it was reported that some phenolic compounds can be liberated from the matrix during the drying process at high temperatures [32].

## 3. Materials and Methods

### 3.1. Chemicals

Authentic standard of rutin and Folin-Ciocalteu reagent were purchased from Sigma-Aldrich (St. Louis, MO, USA). All the used solvents were of HPLC grade purity (BDH Laboratory Supplies, Poole, UK). 4-methylpentan-2-ol used as internal standard and a mixture of linear alkanes (C9–C30) in hexane used for calculating linear retention indexes were from Sigma-Aldrich (St. Louis, MO, USA). Inert gasses (He and N_2_ 99.999% purity) were supplied by SOL gas company (Monza, Italy).

### 3.2. Collection and Drying of Samples

Two different dried white onion (*Allium cepa* L.) samples were purchased in 2017 by Officinali Agribioenergia Factory (Medicina, Bologna, Italy). The two different samples were obtained by different cuts and different drying cycles. For obtaining onion flakes, the fresh onion was cut into cubes of 8 mm sides, which were then dried in a static oven (with low air ventilation, that is, 10000 m^3^/h) for 24 h at 40 °C; onion rings were instead obtained by cutting the fresh onion in order to obtain fresh rings, which were then dried in the same static oven for 31 h at 40 °C. The temperature applied for the drying process were below the 60 °C suggested in previous studies as suitable for avoiding quality losses in the product [33].

The dried samples were deep-frozen with liquid nitrogen and immediately chopped with mortar and pestle, until a fine and homogenous powder was obtained. The powder was used for the following analyses.

### 3.3. Analysis of Phenolic Compounds

A quantity of 250 mg of sample (onion flakes/rings) was extracted in 5 mL of 70% ethanol (pH = 3.2 by formic acid) overnight. The obtained solution was used for the determination of phenolic content by the Folin-Ciocalteu assay and for HPLC-DAD analysis.

#### 3.3.1. Folin-Ciocalteu Assay

The total phenolic content was determined using the Folin-Ciocalteu method [34] slightly modified [35]. In a 15-mL plastic flask, 0.5 mL of deionized water and 125 µL of the Folin-Ciocalteu reagent were added to 125 µL of the suitably diluted sample extract. The mixture was kept for 6 min and then 1.25 mL of a 7% aqueous Na_2_CO_3_ solution were added. The final volume was adjusted to 3 mL with water. After 90 min, the absorption was measured at 760 nm against water as a blank. The amount of total phenolic compounds was expressed as gallic acid equivalents (GAE, mg gallic acid/100 g sample) through the calibration curve of gallic acid. The calibration curve ranged from 20 to 500 µg mL^−1^ (R^2^ = 0.9969).

#### 3.3.2. HPLC-DAD Analysis of Flavonoids

Analysis of the onion extracts was performed using an Agilent 1200 HPLC system (Agilent Technologies, Santa Clara, CA, USA). Samples were separated by a LUNA C18 column (250 × 4.6 mm i.d., 5 µm particle size) maintained at 27 °C. The flow rate was 0.8 mL/min. The mobile phase consisted of (A) water (pH = 3.2 HCOOH) and (B) 100% acetonitrile. The following binary gradient was used: 0–3 min, 5%–12% B; 3–8 min, 12%–14% B; 8–25 min, 14%–20% B; 25–35 min, 20%–35% B; 35–45 min, 35%–60% B, 45–60 min, 60%–100% B. UV/Vis spectra were recorded in the 190–600 nm range and the chromatograms were acquired at 260, 280, 330 and 350 nm. Identification of the quercetin glucosides was based on spectra, standards and literature data, while identification of other individual phenols was carried out using their retention times, spectroscopic and literature data. Quantification of individual phenolic compounds was directly performed by HPLC-DAD using a five-point regression curve (R^2^ ≥ 0.998) in the range 0–30 μg on the basis of quercetin rutinoside standard. Quercetin derivatives were determined at 350 nm using rutin as reference compound. In all cases, actual concentrations of the derivatives were calculated after applying corrections for differences in molecular weight. Three samples were collected from each site so as to express the analytical results as an average with its standard deviation.

### 3.4. Analysis of Volatile Organic Compounds

Volatile organic compounds (VOCs) were analyzed by both HS-SPME-GC-MS and HS-SPME-GC×GC-TOF analyses. Some trials were initially carried out aimed at optimizing sample amount and exposure time and temperature. For both the analyses, after these trials (briefly described in the supplementary file), SPME conditions were set as follow, bearing in mind that we were working with processed samples and also according to Colina-Coca et al. [14]: 15 mg of sample were placed into a 20-mL screw cap vial fitted with PTFE/silicone septa, together with 2 g of NaCl, 5 mL of deionized water and (only for HS-SPME-GC-MS analysis) 5 µL of internal standard (4-methylpentan-2-ol, 20 mg L^−1^ in water). In addition, for HS-SPME-GC-MS, samples of onion flakes and onion rings were also prepared in the same conditions described above and adding 100 mg of ascorbic acid.

After 5 min of equilibration at 60 °C, VOCs were absorbed exposing a 2-cm divinilbenzene/carboxen/polydimethylsiloxane SPME fiber (DVB/CAR/PDMS by Supelco) for 5 min into the vial headspace under orbital shaking at 500 rpm and then immediately desorbed at 280 °C in a gas chromatograph injection port operating in splitless mode. Samples were analyzed in triplicate.

#### 3.4.1. HS-SPME-GC-MS Analysis

The VOCs absorbed as described above were immediately desorbed at 280 °C in the injection port of a 7890a GC system (Agilent Technologies, Santa Clara, CA, USA), separated by a DB InnoWAX column (0.4 µm d.f. × 0.2 mm i.d., 50 m) and detected by a quadrupole Mass Spectrometer 5975c MSD (Agilent Technologies, Palo Alto, CA, USA) operating in EI mode at 70 eV. Initial oven temperature was 40 °C, held for 1 min, then raised to 220 °C at 5 °C min^−1^, then raised to 260 °C at 10 °C min^−1^ and finally held at 260 °C for 4 min. Carrier gas, helium at a flow rate of 1.2 mL min^−1^. Mass spectrometer worked in the mass range 29–350 *m*/*z* and with an electron ionization of 70 eV and the Total Ion Current chromatograms were recorded. Compounds were tentatively identified by comparing the mass spectra of each peak with those reported in mass spectral database as the standard NIST08/Wiley98 libraries, with a minimum matching factor of 80%. Peaks identification was then confirmed by comparing their retention index, calculated by the generalized equation [19] after injecting a mixture of linear alkanes (C9–C30) in hexane (Sigma Aldrich, St. Louis, MO, USA) in the same condition already described for sample analysis, in the literature [36].

Relative concentration of each identified compound was calculated according to previous literature [12], using the following formula:

[VOC (µg/g)] = (A_VOC_/A_ISTD_) × (m_ISTD_ (µg)/m_sample_ (g)),

where A_VOC_ is the area of the VOC, A_ISTD_ is the area of the internal standard, m_ISTD_ and m_sample_ are the amount of internal standard and of sample added into the vial, respectively.

#### 3.4.2. HS-SPME-GC×GC-TOF Analysis

GC×GC was performed by a flow modulation apparatus consisting of an Agilent 7890B GC (Agilent Technologies, Palo Alto, CA, USA), with capillary flow modulator device for 2D separation, coupled with a time-of-flight mass spectrometer (TOF-DS Markes International Ltd., Llantrisant, UK)). SPME sampling was carried out at the same conditions described in 3.4 for mono-dimensional GC-MS analysis. Chromatographic separation was performed using a first dimension (1D) HP-5 column (0.18 μm d.f. × 0.18 mm i.d., 20 m) and an InnoWAX second dimension (2D) column (0.23 μm d.f. × 0.32 mm i.d., 5 m). Flow modulation was performed with a modulation period of 3 s. Helium was used as carrier gas (99.999% purity) at flow rates of 0.4 and 10 mL min^−1^ in first and second dimensions, respectively. The chromatographic conditions were—oven temperature program, 40 °C, increased at 4 °C min^−1^ to 220 °C, increased at 10 °C min^−1^ to 260 °C (hold 1 min); injector temperature, 260 °C; split ratio 1:5. The inlet of the 2D column was maintained under vacuum by a deactivated fused silica (15 cm 0.10 mm i.d.) placed immediately before the column, after the flow modulator. TOFMS parameters—the ion source temperature was 230 °C; the transfer line temperature was 280 °C; ionization, −70 eV. A mass range of 43–500 Da was used, with data rate of 50 Hz. TOF-DS TM software, version 2.0 (Markes International Ltd.; Llantrisant, UK, 2016) was used for data acquisition. GC IMAGE version R2.5 GCGC (64 bit) software (GC IMAGE; LCC-Lincon, Lincoln, NE, USA, 2014) was used for data processing.

Compounds were tentatively identified comparing mass spectra of each peak with those reported in mass spectral databases; identification was confirmed by their retention index.

### 3.5. Statistical Analysis

Quantitative data (Table 1) are expressed as the mean of three determinations. One-way ANOVA and F-test (*p* < 0.05) were applied using Microsoft Excel statistical software for evaluating statistical differences between samples. Fisher’s LSD test was then used for comparing means (DSAASTAT excel^®^ VBA macro, version 1.1, Onofri, A.; Pisa, Italy, 2007).

## 4. Conclusions

This study deals with the characterization of two different types of dried onion, obtained by different cuts of an onion from Emilia Romagna (Italy) dried in a static oven at 40 °C. The flavonoid fraction was analyzed by HPLC-DAD and the volatile fraction was analyzed by both HS-SPME-GC-MS and HS-SPME-GC×GC-TOF, with particular attention to the sulfur volatile compounds. The analysis was mainly focused on characterizing the samples and pointing out any differences in their quali-quantitative volatile and flavonoid profiles. The study also pointed out the presence of some volatile molecules never before reported in the volatile profile of dried onion samples—allyl propyl sulfide, 1-propenyl propyl sulfide, allyl isopropyl disulfide, allyl *cis*-1-propenyl disulfide, allyl *trans*-1-propenyl disulfide, 1-(1-(methylthio)propyl)-2-propyl disulfide, methyl 1-(propylthio)propyl disulfide, 1-(*cis*-1-propenylthio)propyl propyl disulfide, 1-(1-*trans*-propenylthio)propyl propyl disulfide. Some analysis, carried out in the presence of high amount of the antioxidant ascorbic acid, allowed confirming that in the volatile fraction of these dried onion samples, nonoxidized volatile *S*-compounds as thiols are absent or present in negligible amounts. To the author knowledge, this study is the first one about the use of HS-SPME-GC×GC-TOF to deepen the characterization of the volatile fraction of dried onion samples.

The flavonoid content was higher in onion flakes (3.56 mg g^−1^) than in onion rings (2.04 mg g^−1^). At the same time, the total content of volatile compounds was three time higher in onion flakes than in onion rings (127.26 mg g^−1^ vs. 42.79 mg g^−1^), with more than 95% of this amount constituted by sulfur containing compounds. Dipropyl disulfide and dipropyl trisulfide were the most abundant VOCs, with disulfides that largely predominate the volatile fraction (about 60% on the total volatile content in onion flakes and about 77% of the total volatile content in onion rings), followed by trisulfides. HS-SPME-GC×GC-TOF allowed detecting the presence of some molecules not detected by HS-SPME-GC-MS and provided a fast and direct visualization and comparison of different samples. These results highlight different nutraceutical properties of dried onion samples processed otherwise, only differing in shape and size, thus pointing out potentially different uses as functional ingredients.

In light of the obtained results, highlighting different volatile and flavonoid profiles, different uses for the different parts of dried onion can be proposed by dried onion producers to the industry of food ingredients and beyond.

## Figures and Tables

**Table 1:** Relative concentration (µg g^−1^ based on the relative quantitation using internal standard, of volatile organic compounds detected acid by HS-SPME-GC-MS in the different onion samples in the absence and presence of ascorbic acid. Data are the mean of three determinations. Retention Indices (RI_cal_): Non-isothermal Kovats retention indices from temperature-programming, using the definition of Van den Dool and Kratz, 1963 [19]. Retention Indices (RI_ref_): Non-isothermal Kovats retention indices from temperature-programming from Chemistry WebBook. Not dectected (n.d.). traces (tr). For each compound, different letters (a, b, c) indicate significant differences at *p* < 0.05 by Fisher’s Least Significant Difference (LSD) test.

Compound	RI_ref_	RI_cal_	Identification Ions		Dry Onion (µg g^−1^)		Dry Onion with Ascorbic Acid (µg g^−1^)	
					Flakes	Rings	Flakes	Rings
**monosulfides**				**Σ**	**0.32**	**0.09**	**0.25**	**0.09**
		
dimethyl sulfide	729	729	62, 47, 35		0.07 ^b^	0.03 ^a^	0.06 ^b^	0.03 ^a^
allyl propyl sulfide	1137	1113	116, 87, 73		0.20 ^b^	0.04 ^a^	0.16 ^b^	0.04 ^a^
1-propenyl propyl sulfide	-	1138	41, 116, 74		0.05 ^b^	0.02 ^a^	0.03 ^a^	0.02 ^a^
**disulfides**				**Σ**	**76.76**	**32.89**	**75.04**	**41.74**
		
dimethyl disulfide	1105	1081	94, 79, 45		0.14 ^b^	0.03 ^a^	0.11 ^b^	0.02 ^a^
methyl propyl disulfide	1263	1244	80, 122, 43		2.56 ^b^	0.51 ^a^	2.34 ^b^	0.73 ^a^
methyl *cis*-1-propenyl disulfide	1298	1278	73, 120, 45		2.09 ^b^	0.20 ^a^	1.60 ^b^	0.42 ^a^
methyl allyl disulfide	1322	1293	41, 120, 45		0.05 ^b^	0.02 ^a^	0.05 ^b^	0.04 ^a,b^
methyl *trans*-1-propenyl disulfide	1322	1302	73, 120, 45		2.81 ^c^	0.29 ^a^	2.47 ^c^	0.80 ^b^
isopropyl propyl disulfide	-	1331	150, 43, 108		0.04 ^b^	0.01 ^a^	0.03 ^b^	0.01 ^a^
dipropyl disulfide	1413	1391	150, 43, 108		49.34 ^b^	28.53 ^a^	49.77 ^b^	33.35 ^a^
propyl *cis*-1-propenyl disulfide	1450	1427	148, 106, 41		6.69 ^b^	0.61 ^a^	6.05 ^b^	1.38 ^a^
allyl isopropyl disulfide	-	1443	57, 148, 106		0.46 ^c^	0.14 ^a^	0.47 ^c^	0.25 ^b^
propyl *trans*-1-propenyl disulfide	1473	1452	148, 106, 41		11.30 ^b^	1.53 ^a^	10.87 ^b^	3.34 ^a^
allyl *cis*-1-propenyl disulfide	1464	1480	146, 41, 105		0.05 ^b^	0.01 ^a^	0.04 ^b^	0.02 ^a^
allyl *trans*-1-propenyl disulfide	1533	1500	146, 41, 105		0.08 ^b^	0.03 ^a^	0.08 ^b^	0.04 ^a^
1-(1-(methylthio)propyl)-2-propyl disulfide	-	1876	89, 61, 73		0.15 ^ab^	0.07 ^a^	0.19 ^b^	0.18 ^b^
methyl 1-(propylthio)propyl disulfide	-	1985	117, 75, 41		0.44 ^a^	0.65 ^b^	0.48 ^a^	0.82 ^c^
1-(*cis*-1-propenylthio)propyl propyl disulfide	-	2075	115, 81, 73		0.19 ^b^	0.09 ^a^	0.20 ^b^	0.14 ^a,b^
1-(*trans*-1-propenylthio)propyl propyl disulfide	-	2080	115, 81, 73		0.37 ^b^	0.17 ^a^	0.29 ^a,b^	0.20 ^a^
**trisulfides**				**Σ**	**41.08**	**5.30**	**41.69**	**11.21**
		
dimethyl trisulfide	1403	1399	126, 111, 79		6.01 ^c^	0.49 ^a^	7.16 ^c^	1.97 ^b^
methyl propyl trisulfide	1576	1553	154, 112, 43		6.56 ^c^	0.88 ^a^	6.45 ^c^	1.92 ^b^
dipropyl trisulfide	1713	1695	182, 43, 75		23.28 ^b^	3.32 ^a^	22.08 ^b^	5.37 ^a^
allyl propyl trisulfide	1797	1753	115, 180, 73		0.13 ^b^	0.04 ^a^	0.10 ^b^	0.03 ^a^
propyl *trans*-1-propenyl trisulfide	1770	1768	180, 74, 116		5.10 ^c^	0.57 ^a^	5.90 ^c^	1.92 ^b^
**other S-compounds**				**Σ**	**6.08**	**2.45**	**9.09**	**4.83**
		
carbon disulfide	745	716	76, 44, 32		0.07 ^b^	0.02 ^a^	0.03 ^a^	0.02 ^a^
1-propanethiol	845	816	76, 47, 43		0.21 ^a^	0.14 ^a^	0.84 ^c^	0.46 ^b^
2,4-dimethylthiophene	1197	1199	112, 111, 97		0.42 ^b,c^	0.16 ^a^	0.46 ^c^	0.26 ^a,b^
3,4-dimethylthiophene	1253	1264	112, 111, 97		3.57 ^b^	1.16 ^a^	5.80 ^c^	2.55 ^b^
*cis*-3,5-diethyl-1,2,4-trithiolane	1775	1807	180, 74, 151		0.77 ^c^	0.35 ^a^	0.84 ^c^	0.63 ^b^
*trans*-3,5-diethyl-1,2,4-trithiolane	1795	1826	180, 74, 151		1.04 ^c^	0.62 ^a^	1.12 ^c^	0.91 ^b^
**aldehydes**				**Σ**	**1.80**	**1.31**	**3.71**	**3.41**
		
propanal	784	764	58, 29, 28		0.20 ^b^	0.11 ^a^	0.23 ^b^	0.21 ^b^
2-methyl propanal	800	788	72, 43, 41		0.03 ^a^	0.03 ^a^	0.03 ^a^	0.03 ^a^
2-methyl butanal	916	917	41, 86, 57		0.09 ^a^	0.06 ^a^	0.07 ^a^	0.07 ^a^
3-methyl butanal	914	921	44, 71, 45		0.36 ^c^	0.31 ^b,c^	0.21 ^a^	0.23 ^a,b^
pentanal	978	985	44, 86, 58		0.02 ^a^	0.02 ^a^	0.06 ^b^	0.05 ^b^
hexanal	1084	1088	56, 72, 82		0.30 ^b^	0.23 ^a^	0.47 ^c^	0.31 ^b^
2-methyl-2-butenal	1104	1106	84, 55, 39		0.05 ^a^	0.03 ^a^	0.04 ^a^	0.04 ^a^
2-methyl-2-pentenal	1185	1170	98, 41, 69		0.20 ^c^	0.07 ^a^	0.14 ^b^	n.d.
heptanal	1186	1192	70, 86, 96		0.07 ^a^	0.06 ^a^	0.11 ^b^	0.13 ^b^
octanal	1296	1298	84, 100, 110		0.08 ^a^	0.06 ^a^	0.63 ^c^	0.35 ^b^
(*Z*)-2-heptenal	1339	1336	83, 112, 55		0.10 ^b,c^	0.06 ^a^	0.13 ^b,c^	0.08 ^a,b^
nonanal	1394	1402	98, 57, 114		tr	tr	tr	tr
furfural	1471	1470	96, 95, 39		n.d.	0.06 ^a^	1.00 ^b^	1.55 ^c^
decanal	1515	1507	112, 82, 95		0.16 ^a^	0.13 ^a^	0.49 ^c^	0.28 ^b^
benzaldehyde	1529	1542	106, 105, 77		0.14 ^c^	0.08 ^a^	0.10 ^b^	0.08 ^a^
**ketones**				**Σ**	**0.04**	**0.03**	**0.03**	**0.03**
		
6-methyl-5-hepten-2-one	1338	1340	126, 69, 108		0.04 ^a^	0.03 ^a^	0.03 ^a^	0.03 ^a^
**carboxylic acids**				**Σ**	**0.02**	**0.07**	**5.30**	**3.32**
		
hexanoic acid	1838	1846	87, 60, 73		n.d.	n.d.	0.62 ^a^	0.55 ^a^
2-ethyl hexanoic acid	1950	1951	88, 73, 116		n.d.	n.d.	3.26 ^b^	1.85 ^a^
nonanoic acid	2173	2167	73, 158, 129		0.02 ^a^	0.07 ^a^	1.42 ^c^	0.92 ^b^
**other compounds**				**Σ**	**1.16**	**0.65**	**1.10**	**0.79**
		
2-penthylfuran	1236	1235	138, 81, 82		0.05 ^a,b^	0.02 ^a^	0.07 ^b^	0.05 ^a,b^
1-octen-3-ol	1451	1444	57, 72, 99		0.27 ^b^	0.20 ^a^	0.22 ^a^	0.32 ^a^
isopropyl dodecanoate	1832	1835	200, 102, 183		0.27 ^b^	0.17 ^a^	0.29 ^b^	0.19 ^a^
2,2,4-trimethyl-1,3-pentanediol diisobutyrate	-	1885	71, 83, 111		0.57 ^b^	0.26 ^a^	0.52 ^b^	0.23 ^a^
**Total VOCs content**					**127.26**	**42.79**	**136.21**	**65.42**

**Table 2:** Sum of the concentration (µg g^−1^) of the sulfur-containing volatile organic compounds (VOCs) with either the 1-propenyl moiety or the allyl moiety in the onion samples analyzed in the absence of ascorbic acid.

	**1-Propenyl**	**Allyl**
	**(µg g^−1^)**	**(µg g^−1^)**
Flakes	28.73	0.97
Rings	3.52	0.28

**Table 3:** Main volatile organic compounds detected by HS-SPME-GC×GC-TOF in the different onion samples. Data are the mean of three determinations. First dimension retention time in minute (1st D RT). Second dimension retention time in second (2nd D RT). Response of the two-dimensional peak (volume). Relative amount (%) calculations were based on the ratio between the peak volume of each compound and the sum of volumes of all selected compounds. Not detected (n.d.).

Compound	1st D RT (min)	2nd D RT (s)	Rings (volume)	Flakes (volume)	Rings (%)	Flakes (%)
propyl mercaptan	6.05	0.42	1,724,317	2,979,593	5.0	5.8
allylthiol	6.20	0.62	816,521	n.d.	2.4	n.d.
2-methyl-2-butenal	8.65	1.04	677,649	n.d.	2.0	n.d.
2,4-dimethylthiophene	12.85	1.06	562,705	787,731	1.6	1.5
3,4-dimethylthiophene	13.80	1.24	1,170,022	6,773,463	3.4	13.2
methyl propyl disulfide	14.80	1.04	416,658	2,247,811	1.2	4.4
methyl 1-propenyl disulfide	15.15	1.28	149,034	1,541,084	0.4	3.0
dimethyl trisulfide	16.40	1.72	133,334	1,679,770	0.4	3.3
dipropyl dilsulide	21.60	0.94	11,529,581	14,500,819	33.4	28.3
propenyl propyl disulfide	21.95	1.10	1,365,546	3,030,564	4.0	5.9
diethanol disulfide	23.40	1.38	740,611	3,813,741	2.1	7.4
unidentified sulfide mw 152	23.95	1.68	254,301	2,194,284	0.7	4.3
decanal	25.15	0.92	145,059	1,481,756	0.4	2.9
dipropyl trisulfide	29.50	1.08	8,135,894	5,312,160	23.6	10.4
trans-3,5-diethyl-1,2,4-trithiolane	29.90	1.62	1,341,744	220,925	3.9	0.4
cis-3,5-diethyl-1,2,4-trithiolane	30.15	1.64	2,565,177	912,354	7.4	1.8
propenyl propyl trisulfide	30.15	1.38	1,970,506	3,730,800	5.7	7.3
4,6-Diethyl-1,2,3,5-tetrathiolane	38.40	2.22	809,467	n.d.	2.3	n.d.

**Table 4:** Quali-quantitative flavonols content, expressed as mg_rutin_ mg g^−1^. Data are the mean of three independent determination (Standard Deviation < 5%). For the total flavonoid content, different superscript letters (a, b) indicate significant difference at *p* < 0.05.

	RT (min)	UV Absorption (nm)	Onion Flakes	Onion Rings
			(mg g^−1^)	(mg g^−1^)
**flavonoid**				
Quercetin-7,4′-diglucoside	22.3	252–366	0.05	0.04
Quercetin-3,4′-diglucoside	25.2	265–344	1.76	0.94
Isorhamnetin-3,4′-diglucoside	27.3	266–344	0.11	0.01
Quercetin-3-glucoside	33.7	256–350	0.05	0.02
Quercetin-4′-glucoside	36.9	253–365	1.52	0.87
Isorhamnetina-4′-glucoside	38.0	252–366	0.03	0.13
Quercetin	42.9	255–370	0.04	0.04
**Total flavonoid content**			**3.56 ^b^**	**2.04 ^a^**

## Supplementary Materials

The following are available online, file S1: Brief description of preliminary trials aimed at optimizing sample amount, and exposure time and temperature.
