# Supplementary material for: Characterization of Volatile and Flavonoid Composition of Different Cuts of Dried Onion (Allium cepa L.) by HS-SPME-GC-MS, HS-SPME-GC×GC-TOF and HPLC-DAD
Source: Molecules. 2020 Jan 18;25(2):408. doi: 10.3390/molecules25020408 (PMC7024371; doi:10.3390/molecules25020408)
Supplement: Supplementary file 1 [file molecules-25-00408-s001.pdf]

**Brief description of preliminary trials aimed at optimizing sample amount, and exposure time and temperature.**

The first trial was performed analyzing both onion flakes and onion rings, in order to understand which of the two samples was richer in volatiles, thus then using such sample for the next optimization trials. This trial was carried out by weighting 100 mg of samples and adding them into a 20-mL screw cap vial together with 2 g of NaCl and 5 mL of deionized water. After 5 min of equilibration at 60°C, VOCs were absorbed exposing the 2-cm DVB/CAR/PDMS SPME fiber for 15 min into the vial headspace under orbital shaking at 500 rpm, and then immediately desorbed at 280°C into the gas chromatograph injection port.

The results pointed out that onion flakes was richer in VOCs than onion rings and that some VOCs were too concentrated in the headspace in these conditions (see for example the peak of dipropyl disulfide for the onion ring sample in figure S1).

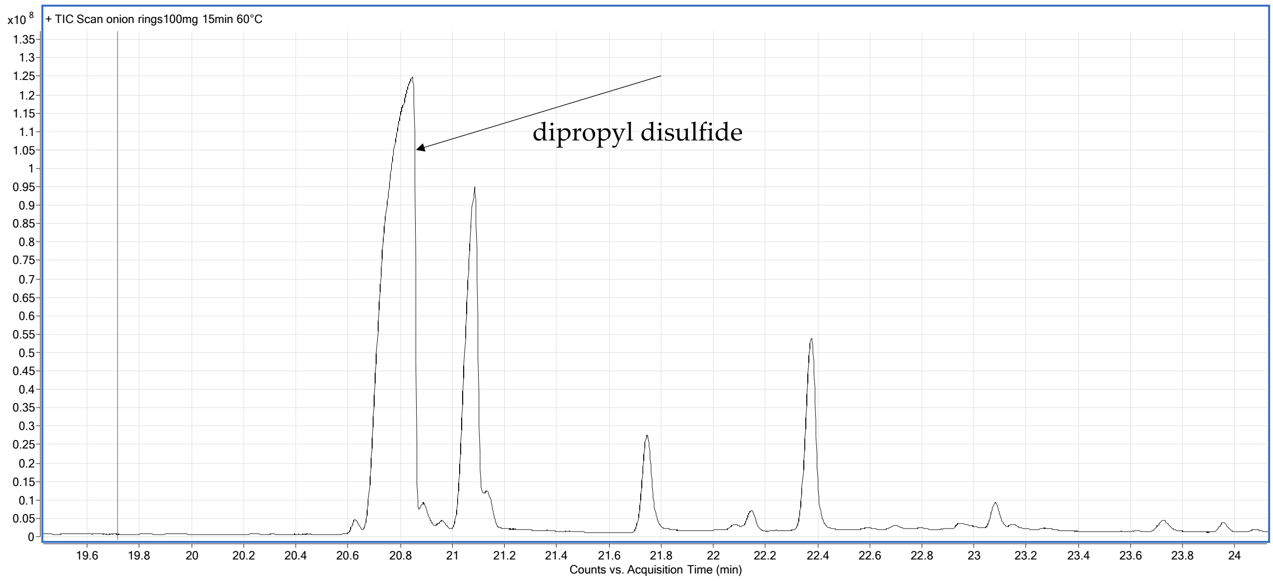


**Figure S1.** TIC of 100 mg onion ring sample adsorbed for 15 min at 60°C.

In the next trials, performed using onion flakes, we immediately reduced absorption time to 5 min. Using this absorption time, we performed some trials weighting 75 mg, 25 mg, 15 mg and 5 mg of sample. This latter amount was considered too low, in that any inhomogeneity of the sample could strongly affect the results, and some peaks were actually too small for detecting them. The trials using 75 mg showed again some peaks in too high amounts, while using 15 mg we obtained the chromatogram reported in figure S2: it showed well-resolved peaks and also suitable intensities for dipropyl disulfide, the most abundant peak. In the last trial, we tried to lower the temperature (30°C) to check any significant influence this change could cause to the profile: only little decrease of area of some peak was observed as expected, due to the lower volatility of the molecules at lower temperature, thus we decided to carry out the analysis using an absorption temperature of 60°C.

The optimized parameters were:

- Sample amount: 15 mg
- Absorption time: 5 min

- Absorption temperature: 60°C

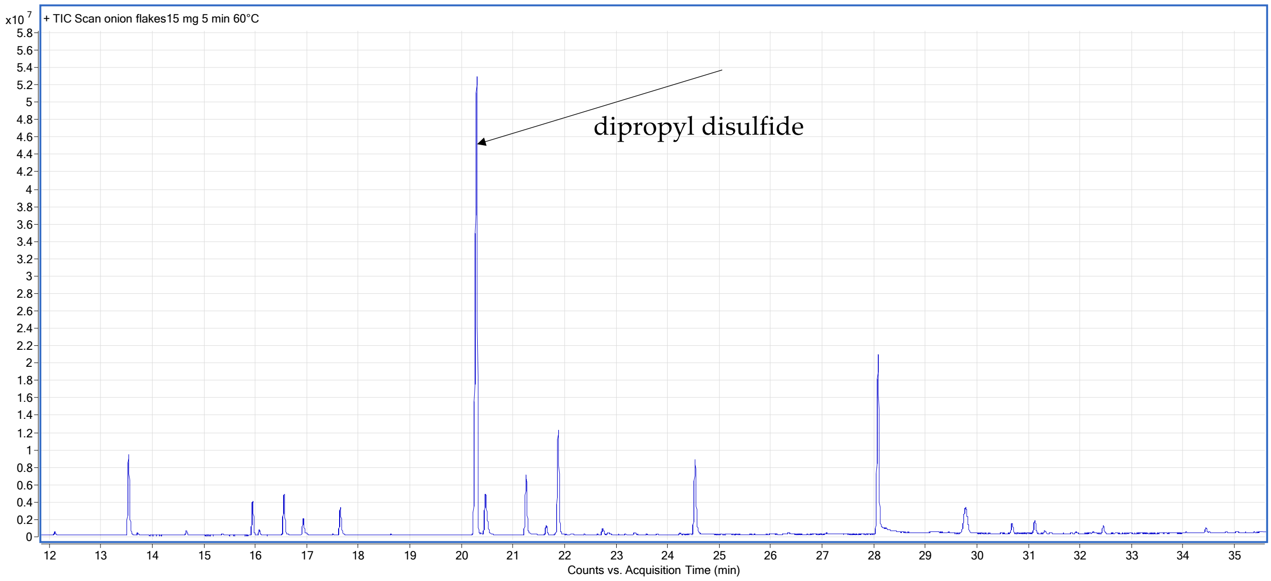


**Figure S2.** TIC of 15 mg onion flakes sample adsorbed for 5 min at 60°C.
